# Protein biomarkers of disease progression in patients with systemic sclerosis associated interstitial lung disease

**DOI:** 10.1038/s41598-023-35840-y

**Published:** 2023-05-27

**Authors:** Giuliana Cerro-Chiang, Matthew Ayres, Alejandro Rivas, Tahmineh Romero, Sarah J. Parker, Mitra Mastali, David Elashoff, Peter Chen, Jennifer E. Van Eyk, Paul J. Wolters, Francesco Boin, Tanzira Zaman

**Affiliations:** 1 Division of Pulmonary and Critical Care Medicine, Cedars Sinai Medical Center, 8700 Beverly Blvd., South Tower Room 6723, Los Angeles, CA 90048 USA; 2 Advanced Clinical Biosystems Institute Biomedical Sciences, The Smidt Heart Institute, Cedars Sinai Medical Center, Los Angeles, CA USA; 3 Division of General Internal Medicine and Health Services Research, University of California Los Angeles, Los Angeles, CA USA; 4 Division of Pulmonary, Critical Care, Allergy, and Sleep Medicine., University of California, San Francisco, San Francisco, CA USA; 5 Division of Rheumatology, Cedars Sinai Medical Center, Los Angeles, CA USA

**Keywords:** Systemic sclerosis, Predictive markers, Respiratory tract diseases

## Abstract

Systemic sclerosis is a rare connective tissue disease; and interstitial lung disease (SSc–ILD) is associated with significant morbidity and mortality. There are no clinical, radiologic features, nor biomarkers that identify the specific time when patients are at risk for progression at which the benefits from treatment outweigh the risks. Our study aimed to identify blood protein biomarkers associated with progression of interstitial lung disease in patients with SSc–ILD using an unbiased, high-throughput approach. We classified SSc–ILD as progressive or stable based on change in forced vital capacity over 12 months or less. We profiled serum proteins by quantitative mass spectrometry and analyzed the association between protein levels and progression of SSc–ILD via logistic regression. The proteins associated with at a *p* value of < 0.1 were queried in the ingenuity pathway analysis (IPA) software to identify interaction networks, signaling, and metabolic pathways. Through principal component analysis, the relationship between the top 10 principal components and progression was evaluated. Unsupervised hierarchical clustering with heatmapping was done to define unique groups. The cohort consisted of 72 patients, 32 with progressive SSc–ILD and 40 with stable disease with similar baseline characteristics. Of a total of 794 proteins, 29 were associated with disease progression. After adjusting for multiple testing, these associations did not remain significant. IPA identified five upstream regulators that targeted proteins associated with progression, as well as a canonical pathway with a higher signal in the progression group. Principal component analysis showed that the ten components with the highest Eigenvalues represented 41% of the variability of the sample. Unsupervised clustering analysis revealed no significant heterogeneity between the subjects. We identified 29 proteins associated with progressive SSc–ILD. While these associations did not remain significant after accounting for multiple testing, some of these proteins are part of pathways relevant to autoimmunity and fibrogenesis. Limitations included a small sample size and a proportion of immunosuppressant use in the cohort, which could have altered the expression of inflammatory and immunologic proteins. Future directions include a targeted evaluation of these proteins in another SSc–ILD cohort or application of this study design to a treatment naïve population.

## Introduction

Systemic sclerosis (scleroderma or SSc) is a rare connective tissue disease characterized by immune dysfunction, vasculopathy, cellular inflammation, and fibrosis. SSc commonly manifests in the skin, lung parenchyma, and pulmonary vasculature^1^. Cohort studies have estimated the prevalence of SSc-related interstitial lung disease (SSc–ILD) at between 30 and 50%^2,3^ with 30–35% of all scleroderma-related deaths attributed to it^4^. SSc–ILD has a variable clinical course^5^. While most patients experience long periods of stability, about a third have disease progression after disease onset that is associated with higher mortality^5–8^.

Given the significant side effects from the existing therapies like immunosuppressant and antifibrotic medications, current guidelines recommend active surveillance and treatment after evidence of progressive lung disease^9,10^, most commonly defined by decline in forced vital capacity (FVC)^11,12^. Risk factors that identify patients at risk for development of ILD do not predict disease progression^1^. Prior studies have determined clinical and laboratory predictors of mortality and progression of ILD, such as presence of the Scl-70 antibody, extent of disease on computed tomography, and decline in FVC and DLCO; however, these do not identify the specific time in the clinical course at which patients are at risk for progression and thus most likely to benefit from initiation of treatment^13,14^. As a result, patients often lose irrecoverable lung function prior to initiation of treatment, as progression is only realized after the fact of its occurrence.

Serum biomarkers of progressive SSc–ILD are an attractive alternative to identify patients at risk for progression given the ease of collection. Prior biomarker studies in SSc–ILD determine the risk of developing ILD but do not predict progression^13,15^, or they have focused on candidate proteins by extrapolating data from other organs affected by systemic sclerosis, such as skin^16–19^. Our study aimed to identify blood protein biomarkers associated with progression of interstitial lung disease in patients with SSc–ILD using an unbiased, high-throughput proteomic approach.

## Methods

### Study design and participants

In this single center, nested case–control study, we identified subjects with SSc–ILD among patients enrolled in the University of California, San Francisco Scleroderma Center registry from September 2015 to June 2020. All patients fulfilled the 2013 American College of Rheumatology/European League Rheumatism criteria for SSc^20^. Presence of ILD was confirmed by radiographic evidence of pulmonary fibrosis on high resolution computed tomography (HRCT) in patients exhibiting forced vital capacity (FVC) < 80% or a greater than 10% decline in FVC (L) over twelve months. Longitudinal pulmonary function testing was used to classify patients having stable SSc–ILD or progressive SSc–ILD: progressive disease cases were identified by a decline in absolute FVC of 5% or more over a 12-month period, whereas stable disease controls were defined as less than 5% change in absolute FVC over 12 months. In cases where patients had multiple sets of PFT measurements that met our definition of progressive disease, we utilized the first set of PFTs that met the definition. The threshold of 5% was chosen based on prior studies in idiopathic pulmonary fibrosis and SSc–ILD in which a decline of FVC of over 5% was associated with higher mortality^7,21–23^, and an analysis from the two largest SSc–ILD clinical trials, which showed that a minimally clinically important difference correlated with an FVC decline of 3%^24^. Cases (SSc–ILD progressors) and controls (SSc–ILD) were matched for sex at birth, age, BMI, and use of immunosuppression at the time of entering the study. All patients included in the analysis had a blood sample available within 3 months of the pre-progression pulmonary function test (PFT) used for classification.

### Specimen processing and liquid chromatography with dual mass spectrometry (LC–MS/MS) analysis

Biobanked serum specimens were utilized for the proteomics study. These samples were collected within three months of disease progression on pulmonary function tests. Peripheral blood was collected from consenting patients at the time of clinical visits. Serum was isolated, aliquoted, and stored at − 80 °C until the time of this study.

Serum samples were depleted of the 14 most abundant proteins using the High Select Top 14 Abundant Protein Depletion Camel Antibody Resin (Thermo Fisher Scientific) to enhance the detection and identification of the less abundant proteins and analyzed as outlined by McArdle et al.^25^. Samples subsequently underwent serum trypsin digestion and desalting prior to tandem liquid chromatography-mass spectrometry analysis. Data-independent acquisition (DIA) analysis was performed on an Orbitrap Exploris 480 (Thermo Fischer Scientific) mass spectrometer interfaced with a microflow-nanospray electrospray ionization source (Newomics, IS-T01) coupled to an Ultimate 3000 ultra-high-pressure chromatography system. Peptides were loaded at 4 µg, based on average bicinchoninic acid assay (BCA) results, and separated on a gradient of 7–10% B organic phase for 2 min, 10–32% B for 53 min, 32–70% for 1 min, 70–70% for 2.5 min, and then decreased from 70% B to 1% B over 1 min, on a C18 column (15 cm length, 300 µm ID, 100 Å pore size, Phenomenex) over the course of 60 min with a constant flow rate of 9.5 µL/min. Mass range of 400–1000 and automated gain control (AGC) target value for fragment spectra of 300% was used. Peptide ions were fragmented at a normalized collision energy of 30%. Fragmented ions were detected across 50 non-overlapping data independent acquisition precursor windows of size 12 Da. MS2 Resolution was set to 15,000 with an ion transmission time of 22 ms. All data is acquired in profile mode using positive polarity. Protein estimates were normalized across batches to mitigate systematic error.

### Statistical analysis

Baseline demographics were described using means, standard deviations, and proportions. For proteins with quantifiable levels in over 50% of the samples, samples with values below the lower level of detection were imputed as one less than the lowest detected value. The protein values were log_10_ transformed for normalization, which was confirmed visually with histograms. When protein levels were detected in less than 50% of the samples, the values were transformed to a dichotomous variable to indicate detected versus not detected.

The association between continuously modelled, log-transformed biomarkers and progression of SSc–ILD was assessed through logistic regression. For proteins that were dichotomized, chi-squared testing was used. The false discovery rate was calculated using the Benjamini Hochberg method.

Principal component analysis was performed to reduce the dimensionality of the protein biomarker set. The relationship between the top 10 principal components and progression was evaluated using logistic regression. Additionally, unsupervised hierarchical clustering by Ward linkage with heatmapping was done to define unique groups.

Least absolute shrinkage and selection operator (LASSO) logistic regression test was performed to construct a protein model for prediction of progressive SSc–ILD. The area under the curve (AUC) for this model was computed to summarize the predictive utility for this combination of biomarkers. A permutation test framework was used to estimate an overall empiric *p* value for the AUC^26^.

Proteins associated with progression of disease at a *p* value of < 0.1 were queried in the QIAGEN Ingenuity Pathway Analysis (IPA) software to identify interaction networks, signaling, and metabolic pathways. The fold change of each protein using the progressive group as the denominator was calculated. The upstream regulator with a predictive activation state or canonical pathways detected with an absolute Z score ≥ 2 were reported.

Additional analyses included logistic regression models to determine the association between proteins and progression of FVC. In addition to unadjusted logistic regression, multivariable regression models adjusting for clinical variables of race, smoking status, systemic sclerosis subtype, presence of pulmonary hypertension, anti-Scl-70 and ACA antibody status, pre-progression FVC (L), and pre-progression DLCO adjusted for hemoglobin (cc/sec/mmHg) were also performed. StataSE 17 software was used for statistical analysis.

Informed consent was obtained from all subjects and all methods and samples were collected and stored per the Institution Review Board approval at the University of California San Francisco. All methods were carried out in accordance with relevant guidelines and regulations.

### Ethical approval

This study was approved by the Internal Review Board of the University of California San Francisco.

## Results

The cohort consisted of 72 patients, 32 of whom had progressive SSc–ILD (cases) and 40 who had stable disease (controls). Baseline characteristics are described in Table 1. In both groups, most patients were women (68.75% and 65%, respectively) and of white race (62.5%). The mean age was 51.6 years and mean BMI was 24 kg/m^2^. The proportion of patients with anti-Scl70 antibody, ACA antibody positivity, and immunosuppression use was similar between groups. There was a higher percentage of never-smokers in the progressive group. Pre-progression absolute and percent-predicted values of FVC and DLCO were lower in the progressive SSc–ILD group. The median percent change in FVC over one year was -18% in the progressive group compared to 4% in the control group. In a multivariable logistic regression model using the clinical variables of race, smoking status, SSc subtype, presence of pulmonary hypertension, anti-Scl-70, and ACA antibody status, pre-progression FVC and pre-progression DLCO were not independently associated with progression of disease.

**Table 1:** Baseline clinical and demographic characteristics of the SSc–ILD cohort.

	Progressive SSc–ILD (n = 32)	Stable SSc–ILD (n = 40)	*p* value
Women, number (%)	22 (68.75)	26 (65)	0.74
Race, number (%)			0.09
White	20 (62.5)	25 (62.5)	
African American	6 (18.75)	1 (2.5)	
Asian	4 (12.5)	10 (25)	
Other	2 (6.25)	4 (10)	
Age, years, mean (SD)	52.9 (12)	50.6 (9.8)	0.37
Anti Scl-70 antibody, number (%)	14 (43.75)	16 (40)	0.75
Anticentromere antibody, number (%)	2 (6.2)	1 (2.5)	0.42
Smoking status, number (%)			0.01
Never	26 (81.25)	23 (57.50)	
Current	2 (6.25)	0	
Former	4 (12.50)	17 (42.50)	
BMI, kg/m^2^, mean (SD)	23.9 (4.8)	24 (5)	0.89
Diffuse SSc, number (%)	10 (31.25)	16 (37.5)	0.45
Modified Rodnan skin score, median [IQR]	3 [3–4]	3.5 [3–6.25]	0.24
Presence of pulmonary hypertension (%)	37.5	15	0.02
Use of immunosuppression, number (%)	31 (96.88)	36 (90)	0.26
Time from first Raynaud’s symptom onset to ILD diagnosis median [IQR]	1.76 [0.27–6.58]	4.37 [1.69–9.43]	0.08
Time from first non-Raynaud’s symptom onset to ILD diagnosis median [IQR]	1.05 [0.21–4.02]	2.40 [0.84–6.26]	0.09
Pre-progression FVC, L, median [IQR]	2.23 [1.96–2.55]	2.77L [2.50–3.04]	0.075
Pre-progression FVC, %predicted, median [IQR]	61% [49.5–69]	72% [60–83]	0.01
Pre-progression DLCO corrected for hemoglobin, mL/min/mmHg, median [IQR]	11.8 [8.7–15.09]	16.5 [12.9–19.4]	0.04
Pre-progression DLCO corrected for hemoglobin, %predicted, median [IQR]	40 [33–58]	58 [44–71]	0.01
Percent change in absolute FVC over 1 year median [IQR]	− 18% [− 27% to 16%]	4% [− 2% to 9%]	< 0.01
Time between blood collection and pre-progression PFT, months, median [IQR]	1 [− 0.52 to 2.1]	0.32 [− 1.15 to 1.43]	0.13

*SSc–ILD* systemic sclerosis ILD, *SD* standard deviation, *BMI* body mass index, *SSc* systemic sclerosios, *IQR* interquantile range, *ILD* interstitial lung disease, *FVC* forced vital capacity, *DLCO* diffusing capacity for carbon monoxide, *PFT* pulmonary function test.

A total of 794 proteins were detected. Of these, 285 proteins had quantified values for all patients. There were 367 proteins for which quantified values were detected for greater than 50% of subjects and for which values below the level of detection were imputed. There were 142 proteins for which greater than 50% of patients’ values were undetected and were thus treated as dichotomous values (detected/undetected).

On unadjusted logistic regression, 29 proteins exhibited an association with ILD progression at greater than 0.05 significance level (Table 2). After adjusting for multiple testing, these associations did not remain statistically significant. Additionally, there were no significant associations between pre- progression FVC and protein levels after adjusting for multiple testing. When adjusted for clinical variables, there were no significant associations between protein levels and progression of disease or pre- progression FVC.

**Table 2:** Protein biomarkers associated with progression of SSc–ILD.

Protein name	UniProt database identifier	Z score	Association with progression	*p* value
DBNL	Q9UJU6	2.85	Positive	0.004
Uncharacterized protein Fragment 2	A0A0J9YY99	2.38	Positive	0.018
RIN3	Q8TB24	2.22	Positive	0.027
PDCD6	O75340	2.17	Positive	0.030
RNASE3	P12724	2.14	Positive	0.032
KRT2	P35908	2.14	Positive	0.032
IGHV3-49	A0A0A0MS15	2.13	Positive	0.033
Uncharacterized protein Fragment 1	A0A0J9YY99	2.06	Positive	0.040
KRT9	P35527	2.05	Positive	0.041
LYZ	P61626	2.04	Positive	0.042
IGKJ3	A0A0A0MT96	2.03	Positive	0.043
LILRA3	Q8N6C8	3.29	Positive	0.044
PRTN3	P24158	1.99	Positive	0.047
CDH2	P19022	− 2.63	Negative	0.008
NEO1	Q92859	− 2.57	Negative	0.010
A1BG	P04217	− 2.43	Negative	0.015
B3GNT2	Q9NY97	− 2.39	Negative	0.017
ZNF500	ZNF500	− 2.27	Negative	0.023
SERPINF2	P08697	− 2.25	Negative	0.024
BTD	P43251	− 2.23	Negative	0.026
KLKB1	P03952	− 2.19	Negative	0.028
PLG	P00747	− 2.18	Negative	0.029
ANPEP	P15144	− 2.11	Negative	0.035
YBX2	Q9Y2T7	− 2.11	Negative	0.035
PON1	P27169	− 2.11	Negative	0.036
HLA− H	P01893	− 2.09	Negative	0.037
FETUB	Q9UGM5	− 2.04	Negative	0.041
BCHE	P06276	− 2.02	Negative	0.044
ENG	P17813	− 1.99	Negative	0.047

**Figure 1:**
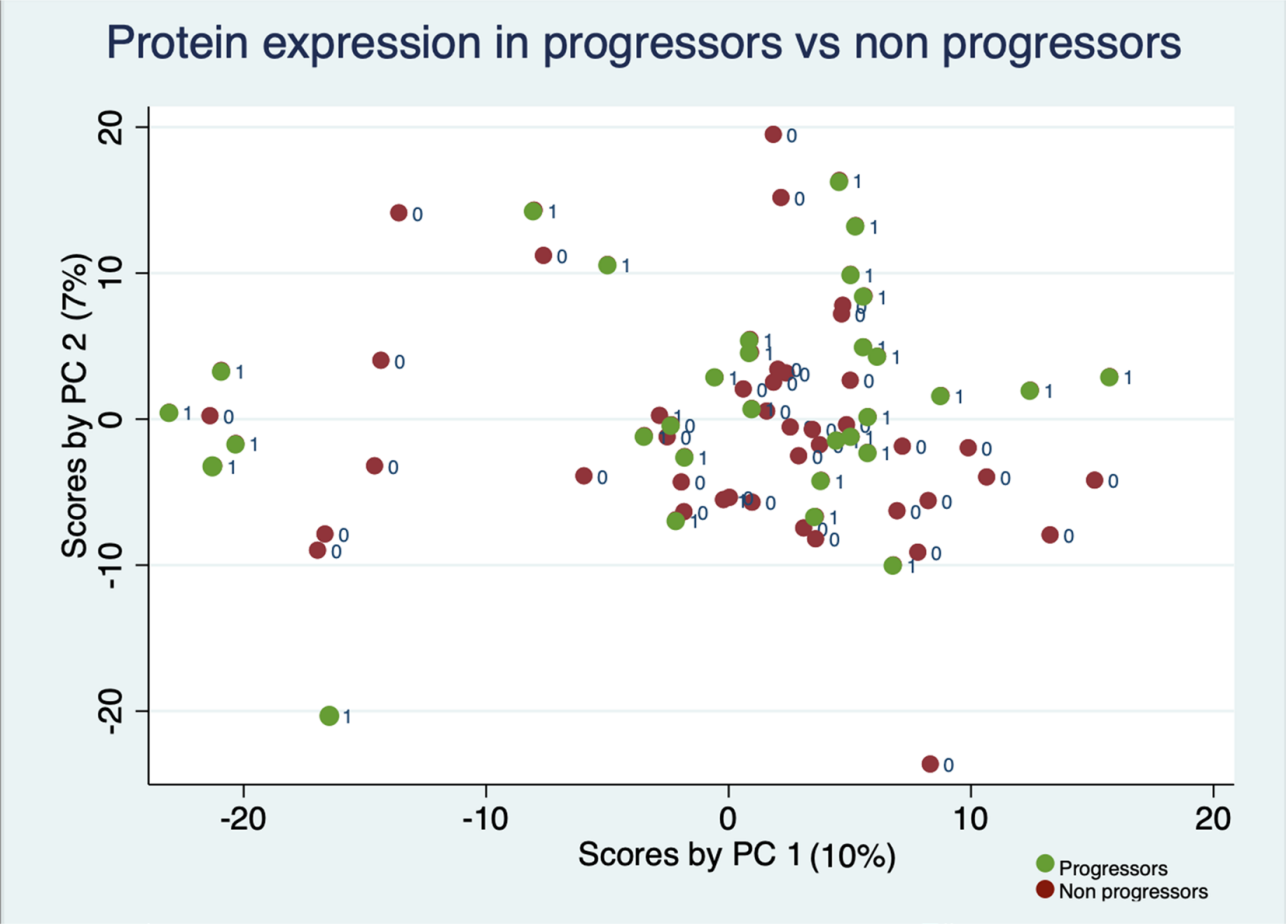
Protein expression in progressors and non-progressors by principal component analysis. (StataSE 17 https://www.stata.com).

**Figure 2:**
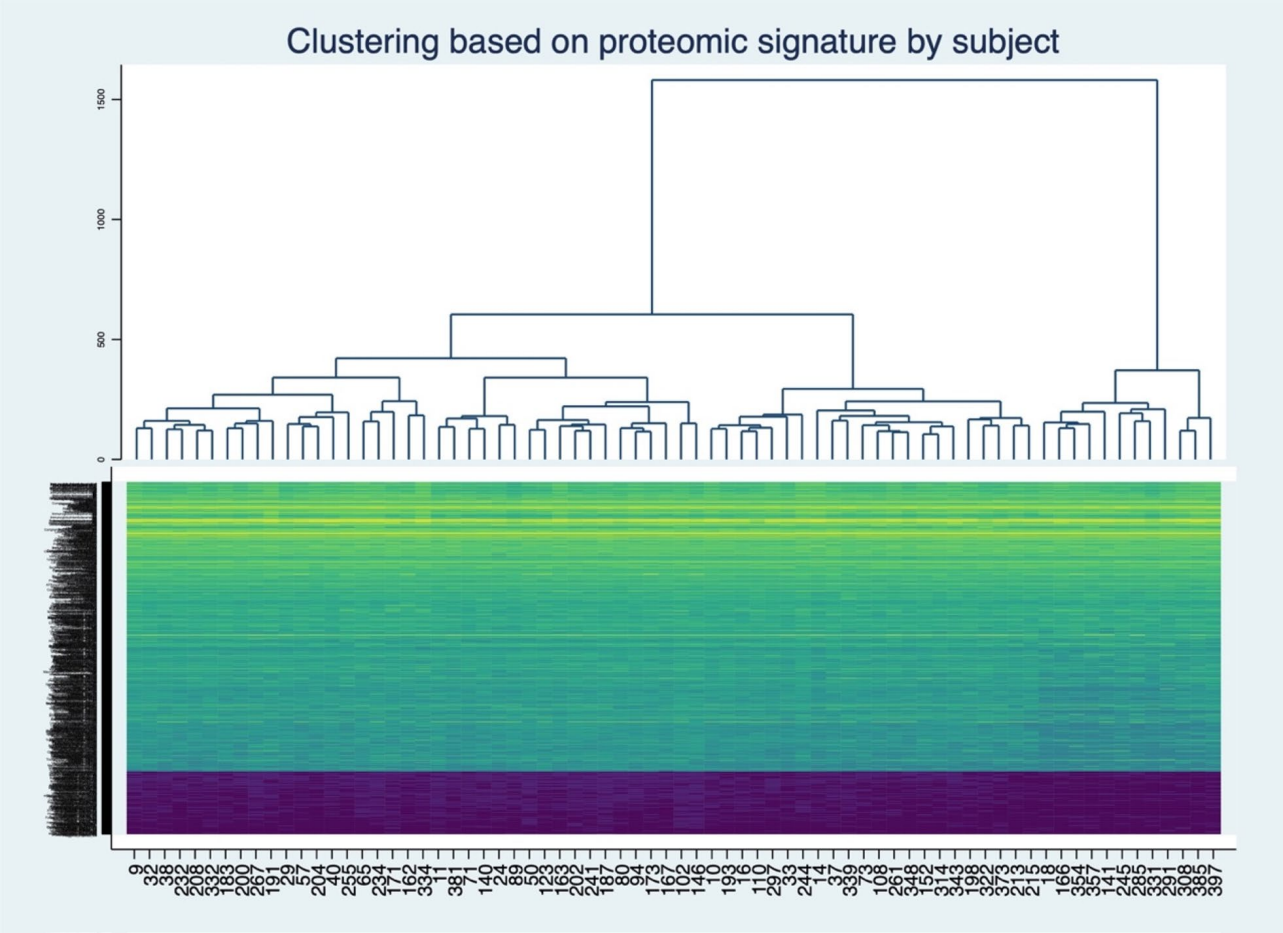
Hierarchical clustering of proteomic data using Euclidean distance and Ward linkage (StataSE 17 https://www.stata.com).

Principal component analysis revealed 72 components, and the ten components with the highest Eigenvalues represented explained 41% of the variability of the sample. There was no significant association between each of these ten components and progression of disease (Table 3). A scatterplot of the two principal components with the highest Eigenvalues shows no differentiation between groups (Fig. 1). Unsupervised clustering analysis revealed no significant heterogeneity between the subjects (Fig. 2).

**Table 3:** Association between principal components and progression of disease.

	Eigenvalue	Proportion	Cumulative	*P* value
Component 1	83.02	0.10	0.10	0.61
Component 2	57.49	0.07	0.17	0.22
Component 3	33.89	0.04	0.22	0.25
Component 4	27.23	0.03	0.25	0.13
Component 5	25.91	0.03	0.29	0.77
Component 6	24.66	0.03	0.32	0.18
Component 7	21.58	0.03	0.35	0.49
Component 8	19.57	0.03	0.37	0.89
Component 9	18.50	0.03	0.39	0.56
Component 10	17.04	0.03	0.41	0.48

A LASSO model identified the DBNL (Drebin like protein) and RIN3 (RIN3 protein, found in many immune cells) proteins, as well as an uncharacterized protein as potential candidates for predictors of progression (Table 4). DBNL and RIN3 have been shown to be autoantigenic in neurologic diseases like encephalitis and schizophrenia respectively^27,28^. Additionally, DBNL, a cytoskeletal protein is involved in T cell signaling and activation^29^. The area under the curve for this model was 0.839 (predicted AUC 0.937), and the empiric p-value for the model was 0.3.

**Table 4:** Odds for progressive SSc–ILD in proteins identified from the LASSO model.

Protein name	OR (95% CI)	*p* value
Uncharacterized protein Fragment 1	6.73 (1.84–24.63)	< 0.001
DBNL	11.97 (3.15–45.42	< 0.001
RIN3	33.51 (4.16–270.05)	0.004

All proteins with a *p* value of < 0.1 were queried in the QIAGEN Ingenuity Pathway Analysis. There were five upstream regulators that targeted molecules in the dataset with an absolute Z score ≥ 2. Of these, three predicted an activated state in the progressive group: rosiglitazone (Z-score 2.173), PKD1 (Z-score 2) and phytohemagglutinin (Z-score 2). Two regulators predicted an inhibited state in the progressive group: EDN1 (Z-score − 2.183) and beta-estradiol (Z score − 2.309). The canonical pathway of liver X receptor/retinoid receptor (LXR/RXR) activation, associated with inflammation and lipid metabolism, had a higher signal in the progression group (Z-score 2.121).

## Discussion

In this study, we identified 29 proteins that were associated with progressive SSc–ILD. While these associations did not remain statistically significant after accounting for multiple testing, these the proteins were found to be part of pathways relevant to autoimmunity and fibrogenesis. We found that the LXR/RXR pathway, which is associated with inhibition of the expression of interleukin-6, cyclooxygenase-2, and nitric oxide synthase^30^, was highly expressed in the progressive group. It suggests that the disease signature is constant between stable and progressors but only that LXR/RXR is activated involved in NO and macrophage signaling in the latter group^31^. Rosiglitazone, a PPAR-γ agonist, has been studied as an attenuator in scleroderma lung fibroblasts^32–34^. The rosiglitazone pathway was more significantly activated in the progressor group. Additionally, the beta-estradiol pathway was more inhibited in the progressor group, which was not explained by sex differences between the two groups. Given prior data showing that men with SSc–ILD have increased ILD severity and higher mortality compared to women^35,36^, this could signal a role of sex hormones in the pathophysiology of fibrotic lung disease.

Currently, there is an unmet need to identify patients at risk for progression of SSc–ILD to optimize timing of treatment initiation. To date, there are no such indicators, and progression is only realized after the fact with decrements in FVC or radiologic findings. Given the ease of collection, blood biomarkers are desirable relative to other sites (e.g. bronchoalveolar lavage fluid or biopsied lung tissue). Prior biomarker studies have focused on establishing the risk for developing ILD but not predicting ILD progression in established disease^37,38^. Our study design, in which we sought to identify a biomarker predicts progression of disease and aids in the crucial clinical decision of when to start therapy for SSc–ILD, is a strength of our study. We utilized blood collected around the time of FVC decline to maximize the likelihood that protein alterations coincided with progression of lung disease rather than abnormalities in other organs affected by systemic sclerosis. Furthermore, our SSc–ILD cohort is well-phenotyped and had longitudinal data to determine disease trajectory. As such, we were able to compare two distinct, clinically relevant trajectories of SSc–ILD.

Sample size was the major limitation of our study, as we had a small number of subjects relative to the large number of proteins queried. Thus, we may have lacked the power to detect statistically significant differences. Similarly, imputation of data for undetected proteins to one less than minimum value may have narrowed the differences in protein levels between the two groups, which would favor the null hypothesis. Additionally, a high percentage of patients in both groups were receiving immunosuppressive agents, which could have altered the expression of inflammatory and immunologic proteins. Consequently, the results may not be representative of treatment naïve patients who may have higher differences in these protein levels or differential protein expression altogether from already-treated patients.

There are several general challenges to the discovery of biomarkers in this setting. Unlike other interstitial lung diseases such as idiopathic pulmonary fibrosis or chronic hypersensitivity pneumonitis, SSc involves multiple organ systems, and given the complexity of the disease, concurrent organ involvement may create greater heterogeneity in circulating molecular markers that makes identifying a protein uniquely associated with ILD more challenging. While an unbiased approach can identify novel proteins of interest, the multiple testing that results necessitates a large sample size (or dramatic differences in protein expression between groups) to reach statistical significance. Given the relative rarity of SSc–ILD, sample size is often limited.

In summary, we sought to identify biomarkers predictive of progressive SSc–ILD. We identified 29 proteins involved in the pathways of the inflammatory and fibrotic cascade that could be associated with disease progression, however, after adjusting for multiple testing these associations did not remain significant. Given that some of these proteins have been found to play a role in inflammatory and fibrotic pathways, future directions may include a targeted evaluation of these 29 proteins as predictors in another SSc–ILD cohort, as well as application of this study design to a treatment naïve population.

## Supplementary Information

Supplementary Information.

## Data Availability

All data generated or analyzed during this study are included in this published article (and its Supplementary Information files).

## Abbreviations

SSc: Systemic sclerosis or scleroderma
ILD: Interstitial lung disease
SSc–ILD: Systemic sclerosis associated interstitial lung disease
IPA: Ingenuity pathway analysis
FVC: Forced vital capacity
HRCT: High resolution computed tomography
BMI: Body mass index
LC–MS/MS: Liquid chromatography with dual mass spectrometry
C: Celsius
DIA: Data-independent acquisition
BCA: Bicinchoninic acid assay
AGC: Automated gain control
LASSO: Least absolute shrinkage and selection operator
AUC: Area under the curve
DLCO: Diffusing capacity for carbon monoxide
SD: Standard deviation
ACA: Anti centromere antibody
